# Balance Performance Is Task Specific in Older Adults

**DOI:** 10.1155/2017/6987017

**Published:** 2017-09-05

**Authors:** Ayelet Dunsky, Aviva Zeev, Yael Netz

**Affiliations:** The Academic College at Wingate, Wingate Institute, Netanya, Israel

## Abstract

Balance ability among the elderly is a key component in the activities of daily living and is divided into two types: static and dynamic. For clinicians who wish to assess the risk of falling among their elderly patients, it is unclear if more than one type of balance test can be used to measure their balance impairment. In this study, we examined the association between static balance measures and two dynamic balance field tests. One hundred and twelve community-dwelling older adults (mean age 74.6) participated in the study. They underwent the Tetrax static postural assessment and then performed the Timed Up and Go (TUG) and the Functional Reach (FR) Test as dynamic balance tests. In general, low-moderate correlations were found between the two types of balance tests. For women, age and static balance parameters explained 28.1–40.4% of the variance of TUG scores and 14.6–24% of the variance of FR scores. For men, age and static balance parameters explained 9.5–31.2% of the variance of TUG scores and 23.9–41.7% of the variance of FR scores. Based on our findings, it is suggested that a combination of both static and dynamic tests be used for assessing postural balance ability.

## 1. Introduction

The ability to control balance is based on the integration of sensory information from the somatosensory, vestibular, and visual systems, which work together with the nervous-muscular system to control body alignment with respect to the environment and to stabilize the body's center of mass during perturbations, with subsequent motor output.

Balance is considered a key component in many activities of daily living, from simple activities such as quiet standing, to more complex activities such as walking while talking or while changing directions [1]. These different tasks require different components of balance ability, which are usually divided into two types of balance: static and dynamic. Static balance is defined as the ability to maintain an upright posture and to keep the line of gravity within the limits of the base of support (i.e., quiet standing) [2]. Dynamic balance is defined as the ability to maintain stability during weight shifting, often while changing the base of support [1].

The ability to control balance deteriorates with age as a result of alternations in the vestibular, visual, somatosensory, musculoskeletal, and central nervous systems [3]. In addition, many older people with balance disorders suffer from orthopedic constraints and cognitive impairments [4]. As a consequence, many people in the elderly population show impairments in stability and balance and thus are subjected to a risk of falling in both static and dynamic situations [4–6].

Detection of the level of impaired postural control is important for the assessment of risk of falling, as well as for the evaluation of proper treatment [7]. Therefore, researchers that study the association between balance and falling, or those who examine the effects of an exercise program on balance, use different balance tests that include static or dynamic aspects [8]. As static and dynamic balance controls are based on the integration and coordination of the same systems, some researchers assess only one type of balance control and consider this assessment is the sole measure of balance ability [5, 9]. However, other researchers assess both types of balance and find different levels of performance between them [7, 10]. This discrepancy led researchers to study the relationship between static and dynamic balance ability. For example, Carter et al. [11] showed moderate correlation (*r* = 0.59) between the two types of balance abilities, Mayson et al. [12] reported a range of correlation values between low to moderate (*r* = 0.19–0.60) for different static and dynamic balance tasks, Shimada et al. [13] found almost no relationships between standing balance tests and dynamic balance control during perturbed walking, and Muehlbauer et al. [14] found no significant correlations between the two types of balance.

Consequently, it is currently unclear to what extent static balance ability and dynamic balance ability among adults are correlated. Thus, for clinicians who wish to assess the risk of falling among their elderly patients, it is unclear if using only one type of balance test is adequate as a measure of their balance impairment. Furthermore, if balance is comprised of various unrelated skills, clinicians should develop training programs that will stimulate all of these skills. In this study, we examine the association between a number of static balance measures and two prevalent dynamic balance field tests.

## 2. Materials and Methods

### 2.1. Participants

One hundred and twenty-three community-dwelling older adults, age 65 and over, volunteered to participate in the study. They were recruited from the general community by ads that appeared on Facebook or were distributed in several sports clubs and in local councils located around the Wingate Institute (where the study was conducted). Inclusion criteria were as follows: being able to perform and complete a maximal exercise test and being involved in habitual physical activity at least once a week for at least three months prior to the commencement of the study. Exclusion criteria included a score of <24 on the Mini Mental State Examination (MMSE) [15] and risk factors of health problems raised by the physician. Based on the decision of the physician, eleven volunteers were excluded (mainly due to cardiovascular risk factors); thus the results reported here represent data from 112 participants. Informed consent was obtained from all participants on a consent form approved by the Institutional Review Board of the Hillel Yaffe Medical Center (Hadera, Israel).

### 2.2. Procedure

Participants visited the laboratory twice in one week. At their first assessment session, participants completed a demographic questionnaire, the Instrumental Activities Daily Living (IADL) questionnaire [16], the short version of the Geriatric Depression Scale (GDS) [17], and the MMSE [15]. The demographic details and mean scores of the clinical assessments are presented in Table 1. Then, two dynamic balance assessments were performed. In the second visit the static balance assessments were performed.

#### 2.2.1. Dynamic Balance Assessments

The Timed Up and Go (TUG) [18], the length of time it takes to stand up from a seated position in a chair, walk three meters, walk around a plastic cone, and return to a seated position, measured in seconds. Lower values indicate better balance ability. The TUG is considered to be a measure of dynamic balance as well as a measure of general mobility, since it represents a sum of transitions of the body, including changes of direction and different heights of the center of mass. The test was performed twice. The better score (lower value) was used for data analysis.

The Functional Reach Test (FR) [19], the distance an individual can reach forward beyond arm's length while maintaining a standing fixed base of support, measured in centimeters. Higher values indicate a longer maximal safe standing forward reach. The test was performed twice. The higher score was used for data analysis.

#### 2.2.2. Static Balance Assessment

The assessment of postural control was carried out by the Tetrax® posturography device (BeamMed Ltd. [Sunlight], Petah Tikva, Israel). The Tetrax consists of two pairs of single piezoelectric sensor platforms that record posturographic right and left heel and toe forces applied to the ground. The analysis is based on the vertical pressure applied via the heels and the toes while standing in an upright position on four plates. Data collection was documented in eight different conditions, each of which reflects the influence of different body systems that affect postural control: (a) normal open position (NO): standing straight, with eyes open; (b) normal closed position (NC): standing straight, with eyes closed; (c) pillows open (PO): standing on pillows, with eyes open; (d) pillows closed (PC): standing on pillows, with eyes closed; (e) head right (HR): standing with the head turned right, with eyes closed; (f) head left (HL): standing with the head turned left, with eyes closed; (g) head back (HB): standing with head tilted backward at a 30-degree angle, with eyes closed; and (h) head forward (HF): standing with head tilted forward about 30 degrees, with eyes closed. For each condition, the participant was instructed to maintain an upright position without moving for 30 seconds [20].

For each condition the Tetrax software computed the following parameters: (1) stability index (ST), the total amount of sway from the four footplates (right and left heels, toes of right and left foot), which was totaled and then divided by the subject's weight. The higher the score, the greater the posture instability. This value correlates strongly with the values “area of sway,” “length of sway,” and “amount of sway” of other investigational systems; (2) the pattern of sway intensities at different frequency ranges, as shown by Fourier spectral analysis of the postural sway waves. Eight frequency band ranges (*F*1–*F*8, in Hz) were computed for evaluation of the power-frequency pattern, as follows:* F*1 = low;* F*2–*F*4 = low-medium;* F*5-*F*6 = medium-high; and* F*7-*F*8 = high frequencies of the Fourier band. Abnormally high values on* F*1 may be related to visual dysfunction. Abnormally high values on* F*2–*F*4 may be related to peripheral vestibular dysfunction. Abnormally high values on* F*5-*F*6 may be related to somatosensory dysfunction, and abnormally high values on* F*7-*F*8 may be related to central dysfunction [20]. A previous study demonstrated reliability of the Tetrax device (ICC_2,1_) with the stability index reported to be 0.850 [21].

### 2.3. Data Analysis

The analysis was performed in two steps. In the first step, associations between static and dynamic balance were calculated using Pearson's correlation coefficient (*r*)  (see Tables 4 and 5). In the second step, a regression model was used to assess the effect of static balance, in each one of the eight conditions, on dynamic balance. Two indicators of dynamic balance were utilized as dependent variables: the TUG scores and the FR scores. The explanatory variables were the parameters calculated by the Tetrax software: ST and Fourier spectral analysis for each of the static balance conditions that had a significant correlations with the dynamic variable. As age is considered a significant explanatory variable for physical ability, it was also entered to the analysis.

## 3. Results

The mean age for all participants was 74.6 years, and as can be seen in Table 1 they were predominantly women (77%). The average BMI represents overweighed values for both genders [22]. The IADL questionnaire showed that all participants were independent, and they showed normal values of cognitive functions based on their MMSE results. Most of them had a high school education. Based on the GDS scores, two participants were suggestive of depression (GDS score > 5) [17]. As for physical activity, women reported exercising about one hour a day on average, while men reported an hour and a half each day.

Results of partial correlations between static and dynamic balance variables for women and for men are presented in Tables 4 and 5, respectively. As can be seen, there are very few parameters that show significant correlations. For women, correlations between dynamic balance and static balance were found mainly with static parameters that are correlated with the vestibular system (frequency ranges of sway intensities* F*2,* F*3, and* F*4, and conditions HR, HL, HB, and HF). For men, the only significant correlations were found between the static balance parameters in eyes-opened conditions and the FR scores, while no significant correlations were found between the static balance parameters and the TUG scores.

Results of the regressions of the static balance parameters for the TUG by gender are presented in Table 2. Based on this analysis, it was found that, for women, age significantly explained 22.1% of the variance of the TUG scores, and adding static balance parameters to the regression revealed explanations between 28.1% and 40.4% of the variance of the TUG scores. The frequency ranges of sway intensities that stood out were* F*2,* F*3, and* F*4, which may be related to peripheral vestibular function. For men, age did not significantly explain the variance of the TUG scores, while static balance parameters explained between 9.5% and 31.2% of the variance of the TUG scores; however only two of the regressions were found to be significant.

Results of the regressions of the static balance parameters for FR by gender are presented in Table 3. Based on this analysis, it was found that for women age did not explain significantly the variance of the FR scores, while static balance parameters significantly explained between 14.6% and 24.1% of the variance of the FR scores, in five postural conditions: NC, PC, HL, HB, and HF. These conditions may be related to peripheral vestibular function, because no vision (eyes were closed) is presented in these conditions. For men, age significantly explained 12.8% of the variance in the FR scores, while age and static balance parameters significantly explained between 23.9% and 41.7% of the variance of the FR scores.

## 4. Discussion

The purpose of the current study was to examine to what extent the ability to control balance in static situations can explain performance in dynamic balance situations. The main finding of the study implies that in general there are low associations between static balance, as measured by a posturography system, and dynamic balance, as measured by two dynamic tests (TUG and FR). This finding is similar to the results of Hrysomallis et al. [23], who found weak associations between static balance and dynamic balance among elite football players, the results of Shimada et al. [13] and Muehlbauer et al. [14], who found low correlations or no correlations between static and dynamic balance abilities among elderly, and the results of Pau et al. [24], who found that static balance and dynamic balance were scarcely correlated among people with multiple sclerosis.

In addition, the low correlations found in the current study are indirectly similar to results of Paillard et al. [25], who examined the influence of a walking program on static and dynamic balance (among other variables) for older adults. They found that the intervention significantly increased dynamic balance but not static balance; it was suggested that different conditions and interventions may influence dynamic balance but not static balance and vice versa.

The low correlation between the two types of balance can be explained to some extent by the different demands that are placed on the control systems. During static postures the body's center of mass moves slowly with small sways, due to small external forces acting upon the body, while during dynamic tasks, greater external forces are present and more changes in the environment occur, thus placing higher demands on all balance control systems [23, 26].

The second finding of the current study was that, for women, static balance parameters that are correlated with peripheral vestibular function explained, to some extent, the variance in performance on the TUG assessment. This finding can be justified by the fact that the TUG test is based on changing heights and directions of the body, changes that irritate both the semicircular canals and the otoliths of the vestibular organ. These irritations may appear during head position changes that occur during several conditions of the Tetrax stability test (i.e., the HR, HL, HB, and HF). It is possible that people with low sensitivity to head position changes, as a consequence of vestibular deterioration, may exhibit poor performance of tasks that include head and body position changes. As falls are known to occur most frequently during walking or during transitions from sitting/standing to walking when head acceleration is higher, reduced capabilities of the vestibular system may be the responsible system for falls [27]. Indeed, it was found that vestibular fault (such as symptoms of true vertigo and sensation of movement) is associated with higher risk of falling during simple tasks, such as rising up from a chair or changing body position [9].

It is worth mentioning that our study is based on a group of highly educated volunteers, relatively healthy and active, who may not represent the general population of older adults. On the other hand, the fact that they are healthy and active enables the assessment of balance without the potential moderating effects of diseases and/or medications. Furthermore, our results corroborate previous studies reporting low associations between static and dynamic balance in elderly populations [13, 14]. It is therefore likely to assume that the conclusions of the current study reflect healthy older adults in general.

As many clinicians want to assess the risk of fall among the elderly, a combination of instrumental tests that includes both static and dynamic tasks, as well as questionnaires, should be used, in order to increase the accuracy of such predictions [1, 7, 23]. In addition, as it is widely recognized that older people suffer from a number of impairments [4], physical trainers or physical therapists should include different aspects of balance control, including both static and dynamic exercises, and treat individuals in accordance with their specific impairments [8]. In addition, based on the second finding of the current study, trainers should pay great attention to the performance of their patients during activities that involve direction or height changes.

## 5. Conclusions

Based on the poor associations between static and dynamic balance found in the current study, it should be noted that essential information might be overlooked if only one component is measured. This may be particularly relevant to prospective longitudinal studies investigating the relationship between balance ability and the risk of falling among the elderly.

In addition, for preservation and rehabilitation purposes, physical activity trainers should consider the inclusion of both static and dynamic balance exercises in training programs for older adults.

## Figures and Tables

**Table 1:** Demographic and clinical description of the participants (mean ± standard deviations).

	Women	Men
*N*	86	36
Age (years)	74 (6)	77 (5)
Weight (kg)	68.43 (10.74)	77.89 (9.74)
Height (m)	1.58 (0.06)	1.72 (0.06)
BMI (kg/m^2^)	27.43 (3.98)	26.36 (3.50)
MMSE	29.15 (1.30)	28.69 (1.83)
GDS	2.27 (2.47)	1.67 (2.04)
Education (years)	12.85 (3.31)	14.03 (3.17)
Lawton	1.12 (0.20)	1.08 (0.22)
Physical activity (total min/week)	429.76 (218.63)	577.94 (256.78)

**Table 2:** Regression of static balance parameters on TUG, by gender.

Static balance condition	Women		Men	
	*R* ^2^ total	Variable included	*R* ^2^ total	Variable included
NO	31.7	Age^*∗∗*^, *F*3^*∗*^	9.5	
NC	32.5	Age^*∗∗*^, *F*4^*∗*^	29.6	
PO	33.2	Age^*∗∗*^	14.5	
PC	31.1	Age^*∗∗*^, *F*8^*∗*^	31.2	*F*3^*∗*^
HR	40.4	Age^*∗∗*^, *F*2^*∗∗*^, *F*4^*∗*^	29.5	*F*7^*∗*^
HL	28.1	Age^*∗∗*^	20.4	
HB	34.2	Age^*∗∗*^, *F*2^*∗*^	16.3	
HF	30.2	Age^*∗∗*^, *F*8^*∗*^	26.5	

^*∗*^ *p* < 0.05; ^*∗∗*^*p* < 0.01. NO: normal open position: standing straight, with eyes open. NC: normal closed position: standing straight, with eyes closed. PO: pillows open: standing on pillows, with eyes open. PC: pillows closed: standing on pillows, with eyes closed. HR: head right: standing with the head turned right, with eyes closed. HL: head left: standing with the head turned left, with eyes closed. HB: head back: standing with head tilted backward at a 30-degree angle, with eyes closed. HF: head forward: standing with head tilted forward about 30 degrees, with eyes closed. *F*1–*F*8 represent the pattern of sway intensities at different frequency ranges, as shown by Fourier spectral analysis of the postural sway waves. *F*1 = low, *F*2–*F*4 = low-medium, *F*5-*F*6 = medium-high, and *F*7-*F*8 = high frequencies of the Fourier band.

**Table 3:** Regression of static balance parameters on FR, by gender.

Static balance condition	Women		Men	
	*R* ^2^ total	Variable included	*R* ^2^ total	Variable included
NO	6.8		37.2	Age^*∗*^
NC	22.0	*F*4^*∗*^, *F*8^*∗∗*^	41.7	Age^*∗*^, *F*4^*∗*^
PO	6.9		27.4	Age^*∗*^
PC	24.1	*F*1^*∗*^, *F*2^*∗*^, *F*7^*∗∗*^	23.9	Age^*∗*^
HR	13.7		19.3	Age^*∗*^
HL	16.1	*F*4^*∗∗*^, *F*5^*∗*^	35.2	Age^*∗*^, *F*4^*∗*^
HB	14.6	*F*4^*∗∗*^	35.0	Age^*∗*^
HF	22.8	*F*6^*∗*^	21.3	Age^*∗*^

^*∗*^ *p* < 0.05; ^*∗∗*^*p* < 0.01. NO: normal open position: standing straight, with eyes open. NC: normal closed position: standing straight, with eyes closed. PO: pillows open: standing on pillows, with eyes open. PC: pillows closed: standing on pillows, with eyes closed. HR: head right: standing with the head turned right, with eyes closed. HL: head left: standing with the head turned left, with eyes closed. HB: head back: standing with head tilted backward at a 30-degree angle, with eyes closed. HF: head forward: standing with head tilted forward about 30 degrees, with eyes closed. *F*1–*F*8 represent the pattern of sway intensities at different frequency ranges, as shown by Fourier spectral analysis of the postural sway waves. *F*1 = low, *F*2–*F*4 = low-medium, *F*5-*F*6 = medium-high, and *F*7-*F*8 = high frequencies of the Fourier band.

**Table 4:** Partial correlation between static and dynamic parameters for women, adjusted for age.

Static variables	Dynamic variables	
	TUG	FR
NO *F*1	.092	.022
NO *F*2	.106	.076
NO *F*3	−.113	.040
NO *F*4	.163	.017
NO *F*5	.125	−.025
NO *F*6	.172	−.132
NO *F*7	.070	−.064
NO *F*8	−.114	.049
NO ST	.160	−.082
NC *F*1	−.078	.169
NC *F*2	−.057	.000
NC *F*3	.209	.056
NC *F*4	.289^*∗*^	−.215
NC *F*5	.098	−.021
NC *F*6	.073	−.016
NC *F*7	.014	−.056
NC *F*8	−.143	.212
NC ST	.092	−.110
PO *F*1	−.051	.061
PO *F*2	.159	.006
PO *F*3	.278^*∗*^	−.017
PO *F*4	.187	−.028
PO *F*5	.038	−.139
PO *F*6	.103	−.033
PO *F*7	−.075	−.148
PO *F*8	−.125	.018
PO ST	.105	−.157
PC *F*1	−.045	−.183
PC *F*2	.009	−.004
PC *F*3	.097	−.179
PC *F*4	−.030	−.101
PC *F*5	.105	−.097
PC *F*6	.148	−.084
PC *F*7	.041	−.310^*∗*^
PC *F*8	.309^*∗*^	−.131
PC ST	.069	−.185
HR *F*1	.005	.003
HR *F*2	.227^*∗*^	−.108
HR *F*3	.234^*∗*^	−.090
HR *F*4	.215	−.273^*∗*^
HR *F*5	−.029	−.191
HR *F*6	−.073	−.157
HR *F*7	.039	−.282^*∗*^
HR *F*8	.042	−.118
HR ST	.043	−.221
HL *F*1	.155	−.047
HL *F*2	.145	.013
HL *F*3	.083	−.077
HL *F*4	.234^*∗*^	−.216
HL *F*5	.159	−.014
HL *F*6	.039	−.102
HL *F*7	.030	−.186
HL *F*8	.011	.156
HL ST	.112	−.206
HB *F*1	.116	.014
HB *F*2	.236^*∗*^	−.017
HB *F*3	.015	−.028
HB *F*4	.213	−.223
HB *F*5	.172	−.083
HB *F*6	.096	.032
HB *F*7	.090	−.113
HB *F*8	.259^*∗*^	−.086
HB ST	.119	−.126
HF *F*1	.073	.113
HF *F*2	.162	−.043
HF *F*3	.102	−.162
HF *F*4	.139	−.282^*∗*^
HF *F*5	.075	−.333^*∗*^
HF *F*6	−.002	−.059
HF *F*7	.072	−.344^*∗*^
HF *F*8	−.180	−.012
HF ST	.115	−.268^*∗*^

^*∗*^ *p* < 0.05.

**Table 5:** Partial correlation between static and dynamic parameters for men, adjusted for age.

Static variables	Dynamic variables	
	TUG	FR
NO *F*1	.010	.295
NO *F*2	.107	.027
NO *F*3	.043	−.048
NO *F*4	−.069	.366^*∗*^
NO *F*5	.063	.328
NO *F*6	.022	.407^*∗*^
NO *F*7	.010	.229
NO *F*8		
NO ST	.070	.344^*∗*^
NC *F*1	−.173	.336
NC *F*2	−.214	.141
NC *F*3	.146	.207
NC *F*4	.238	−.122
NC *F*5	−.006	.191
NC *F*6	−.076	.018
NC *F*7	−.044	.186
NC *F*8	−.072	.131
NC ST	.046	.104
PO *F*1	.165	−.035
PO *F*2	.052	−.024
PO *F*3	.030	−.167
PO *F*4	.083	−.203
PO *F*5	.077	−.271
PO *F*6	.181	−.224
PO *F*7	.292	−.338
PO *F*8		
PO ST	.191	−.419^*∗*^
PC *F*1	−.030	.241
PC *F*2	.053	.191
PC *F*3	−.265	.072
PC *F*4	.003	−.052
PC *F*5	−.071	.065
PC *F*6	.098	.070
PC *F*7	.148	.056
PC *F*8	−.011	.096
PC ST	.079	.018
HR *F*1	.174	−.016
HR *F*2	−.072	−.038
HR *F*3	.016	.120
HR *F*4	.012	−.016
HR *F*5	−.124	−.011
HR *F*6	−.062	−.088
HR *F*7	.223	−.148
HR *F*8	−.045	−.043
HR ST	.058	−.135
HL *F*1	.036	.213
HL *F*2	−.090	.140
HL *F*3	−.139	.102
HL *F*4	−.173	−.041
HL *F*5	−.004	−.063
HL *F*6	−.054	−.084
HL *F*7	.027	.026
HL *F*8		
HL ST	−.047	−.039
HB *F*1	−.123	.325
HB *F*2	−.004	.321
HB *F*3	.054	.086
HB *F*4	.094	.185
HB *F*5	.138	.221
HB *F*6	.014	.166
HB *F*7	.086	.206
HB *F*8	−.065	.203
HB ST	.069	.202
HF *F*1	−.096	.158
HF *F*2	.026	.183
HF *F*3	.024	.113
HF *F*4	−.159	.200
HF *F*5	.035	.254
HF *F*6	−.048	.194
HF *F*7	.074	.181
HF *F*8	−.029	−.086
HF ST	.009	.218

^*∗*^ *p* < 0.05.
